# Comprehensive Analysis of Prognostic Alternative Splicing Signatures in Oral Squamous Cell Carcinoma

**DOI:** 10.3389/fonc.2020.01740

**Published:** 2020-08-28

**Authors:** Ruoyan Cao, Jiayu Zhang, Laibo Jiang, Yanting Wang, Xianyue Ren, Bin Cheng, Juan Xia

**Affiliations:** ^1^Hospital of Stomatology, Sun Yat-sen University, Guangzhou, China; ^2^Guangdong Provincial Key Laboratory of Stomatology, Guangzhou, China; ^3^Guanghua School of Stomatology, Sun Yat-sen University, Guangzhou, China

**Keywords:** oral squamous cell carcinoma, alternative splicing, splicing factor, prognosis, Bioinformatics

## Abstract

**Background:**

Alternative splicing (AS) plays an essential role in tumorigenesis and progression. This study aimed to develop a novel prognostic model based on the AS events to obtain more accurate survival prediction and search for potential therapeutic targets in oral squamous cell carcinoma (OSCC).

**Methods:**

Seven types of AS events in 326 OSCC patients with RNA-seq were obtained from the TCGA SpliceSeq tool and the TCGA database. Cox analysis, the least absolute shrinkage and selection operator Cox regression and random forest were employed to establish prognostic models. Genomics of Drug Sensitivity in Cancer (GDSC) was adopted to estimate the possible drug sensiticity. Prognostic splicing factor (SF)-AS network was constructed by Cytoscape.

**Results:**

The final model included 12 AS events, showing satisfactory performance. The area under the curve for 3- and 5-year survival in the training cohort was 0.83 and 0.82, respectively while that in internal validation was 0.83 and 0.82 accordingly. The calibration curve also indicated a satisfactory agreement between the observation and the predictive values. Low-risk patients stratified by the final model presented higher sensitivity to three chemo drugs. Besides, the prognostic SF-AS regulatory network contained five key SFs and 62 AS events.

**Conclusions:**

We developed a powerful prognostic AS signature for OSCC and deepened the understanding of SF-AS network regulatory mechanisms. Low-risk patients tended to be more sensitive to the three chemo drugs while five key SFs including CELF2, TIA1, HNRNPC, HNRNPK, and SRSF9 were identified as potential prognostic biomarkers, which may offer new prospects for effective therapies of OSCC.

## Introduction

Oral squamous cell carcinoma (OSCC), the most common type of head and neck squamous cell carcinoma (HNSCC), exhibits local invasion, early lymph node metastasis and poor prognosis (1). Despite significant advancements in treatment for OSCC, its 5-year overall survival (OS) remains barely changed at approximately 50% (2). Therefore, there is a critical clinical need to understand the disease process, and to come up with more personalized treatment plans to improve the clinical outcomes. Exploration of diverse prognostic models may provide references for the decision-making process of clinicians and there have been emerging prognostic models to implement this idea. We also developed a 3-mRNA signature to predict the survival of OSCC and this published predictive signature outperformed most existing models in prognostic values (3). Nevertheless, with the updating unveiling of the unexploited mechanisms in OSCC, there is still plenty of room for developing novel predictive models based on different features with more comprehensive functions and higher accuracy.

Alternative splicing (AS) is an important post-transcriptional process that occurs in more than 95% of all human genes (4). The specific types of AS include mutually exclusive exons (ME), exon skipping (ES), retained intron (RI), alternative terminator (AT), alternative promoter (AP), alternative acceptor site (AA), and alternative donor site (AD), all of which can result in mRNA isoforms translation and protein diversity with distinct molecular functions. Accumulating evidence indicates that dysregulation of AS is associated with cancer biology, including cell proliferation, invasion, apoptosis and susceptibility to different chemotherapeutic drugs (5). In practical terms, AS signatures of several cancers did perform well as expected and their area under the curve (AUC) were all more than 0.85 (6–8). Thus, it prompts us to postulate that prognostic models based on AS in OSCC might possess favorable prediction value and could have the potential of identifying the sensitivity of patients to chemo drugs.

Furthermore, AS events are intricately regulated by limited splicing factors (SFs). Dysregulation of SFs may result in global changes of cancer-specific AS events and thereby affecting tumorigenesis, development as well as the response to chemotherapy (9–11). Therefore, a comprehensive analysis of the SF-AS network might promote the unraveling of the underlying molecular mechanisms in OSCC oncogenesis and progression. Moreover, it could also facilitate the discovery of novel biomarkers and potential therapeutic targets.

On these foundations, we constructed and validated a prognostic model with satisfactory performances for 3- and 5-year OS based on the AS events in OSCC collected from the Cancer Genome Atlas (TCGA) database. This AS signature stratified these OSCC patients into the high- and low-risk groups while low risk patients tended to be more sensitive to the 3 chemo drugs. Besides, the prognostic SF-AS regulatory network identified 5 key SFs and 62 AS events.

## Materials and Methods

### Data Acquisition and Pre-processing

The TCGA SpliceSeq tool^1^ provides the seven types of AS events mentioned before (ME, ES, RI, AT, AP, AA, and AD) and also offered the quantification of AS events going from zero to one using the percent spliced In (PSI) index (12). The including criteria of AS events in this analysis were PSI values >75% and standard deviation >0.01. A total of 31 normal controls and 326 OSCC patients were enrolled in the AS events, while 32 normal samples and 328 OSCC samples were included in the RNA-seq data set. After filtering out OSCC patients followed for less than 30 days, we eventually included 31 normal controls and 320 OSCC samples in this analysis.

### Cancer Specific AS Events in OSCC

To identify dysregulated AS events in OSCC, we compared the PSI values between normal and OSCC samples using the Wilcoxon rank sum test. False discovery rate (FDR) based on the Benjamini–Hochberg procedure was employed for multiple testing correction of the *P*-value (13). FDR < 0.01 was considered to represent a statistically significant difference. The coactions among the seven types of AS were clearly demonstrated by the UpSet package of R software.

### Construction and Evaluation of AS Prediction Model

The cancer specific AS events identified above were further filtered by univariate Cox analysis which can estimate the association between the PSI values and the OS of patients. Subsequently, these initially selected prognostic AS events were tested using the bootstrapping method to pick out those with more robust prognostic value. To be specific, 70% patients were randomly extracted from the training cohort to evaluate the prognostic value of the initially selected AS events in 1000 iterations. Alternative splicing events with *P* < 0.05 for over 700 times were considered as robust prognostic AS events (14). Given that 132 patients died in our OSCC cohort, it is recommended that less than 13 AS events should be included in the constructed model based on the “EPV (events per variable) 1 to 10 rule of thumb” (15, 16). Hence, the stepwise multivariate Cox regression analysis was conducted to establish AS-derived prognostic models for each type of AS based on the Akaike information criterion (AIC). The least absolute shrinkage and selection operator (LASSO) Cox method based on the 10-fold cross-validations was performed prior to the above multivariate Cox analysis to reduce variables if needed. Ultimately, a prognostic model based on every single type of prognostic AS event was constructed utilizing random forest analysis and multivariate Cox analysis. Random forest was used to select variables based on the “vh” method in the “randomForestSRC” R package.

We also generated a nomogram to predict the individual’s OS at 3-year and 5-year. To assess the performance of the predicted model, we performed the time-dependent receiver operating characteristics (ROC) curve based on “timeROC” R package and obtained the calibration plot as well as Brier score based on the “riskRegression” R package. Brier score calculated above from 0 to 1 was used to quantify the overall performance of the model, and a lower score indicated better performance.

### Internal Validation of the AS Prediction Model

The internal validation of the AS prediction model was achieved by bootstrap resampling (*n* = 1000) recommended in small sample data set (16). The prognostic model was refitted in each resampling and tested on the original study sample to calculate the difference between resampling AUC/Brier score and original AUC/Brier score. The average of all these calculated AUC/Brier score differences presented the optimism in the apparent AUC/Brier score of the prognostic model that was initially developed in the original sample, and the optimism here indicated the level of model overfitting (15, 17). In addition, another method of internal validation (5-fold cross-validation of 1000 repetitions) was conducted.

### The AS-Signature as an Independent Prognostic Factor

Univariate and multivariate Cox proportional hazards models were applied to estimate the hazard ratios (HRs) and 95% confidence intervals (CIs) for the risk of OSCC mortality. A stratified multivariate Cox regression analysis based on the risk status was also performed.

### Clinical Drug Response Prediction

The prediction of chemotherapeutic response for each patient was made by the final AS model based on Genomics of Drug Sensitivity in Cancer (GDSC)^2^ cell line data set using the R package “pRRophetic”. This R package could evaluated the half-maximal inhibitory concentration (IC50) of the included drugs via ridge regression and the accuracy of the prediction was judged via 10-fold cross-validation based on the GDSC training set (18, 19). FDR < 0.05 was considered statistically significant.

### Splicing Factor Genes and the Underlying Regulatory Network

Splicing factors can influence the selection of exon and splicing site, which contributes to the regulation of AS events. Hence, certain SFs may regulate prognosis-related AS events to some extent. We extracted SFs from the SpliceAid 2^3^ database and collected level 3 RNA sequencing data of OSCC available at TCGA data portal^4^. Univariate Cox regression analysis and survival analysis were employed to identify survival-associated SFs. Spearman correlation test was used to select potential regulatory relationships between the survival-related SFs and the survival-related AS events. FDR < 0.05 was considered as cut-off criteria. Finally, we adopted Cytoscape to visualize the regulatory network. Unless otherwise stated, *P* < 0.05 was considered statistically significant. All statistical analyses were performed using R (version: 3.6.2).

## Results

### Overview of AS Events in OSCC Cohort

**FIGURE 1:**
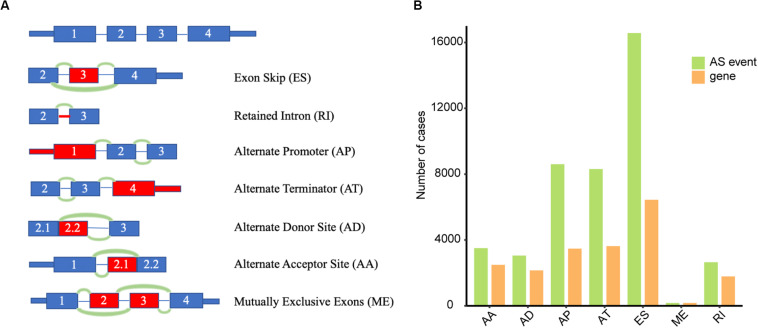
Overview of AS events in TCGA OSCC dataset. **(A)** Illustrations of seven types of AS events, including exon skip (ES), retained intron (RI), alternate promoter (AP), alternate terminator (AT), alternate donor site (AD), alternate acceptor site (AA), and mutually exclusive exons (ME). **(B)** Numbers of AS events and corresponding genes for 320 OSCC patients.

Seven types of AS events, including ES, ME, RI, AP, AT, AD, and AA, were illustrated in Figure 1A. Integrated AS events profiles were analyzed in depth for 320 OSCC patients from the TCGA. A total of 42,849 AS events were detected from 10,123 genes, which suggested one gene could relate to nearly four AS events. More concretely, we identified 16,572 ESs in 6439 genes, 2647 RIs in 1783 genes, 8598 APs in 3469 genes, 8309 ATs in 3627 genes, 3049 ADs in 2148 genes, 3500 AAs in 2484 genes and 174 MEs in 172 genes (Figure 1B). As can be seen, the most common type belonged to ES with a proportion of more than one-third while the minimum proportion went to ME.

### Analysis of Cancer-Specific mRNA Splice Variants

To screen out cancer-specific AS events, we compared the PSI values between the OSCC patients and the normal controls. A total of 5063 events in 2864 genes were dysregulated, including 1376 ESs in 1021 genes, 360 RIs in 302 genes, 1367 APs in 767 genes, 1447 ATs in 826 genes, 258 ADs in 238 genes, 241 AAs in 232 genes and 14 MEs in 14 genes (Supplementary Table S1).

### Construction and Evaluation of the AS Prediction Model

**FIGURE 2:**
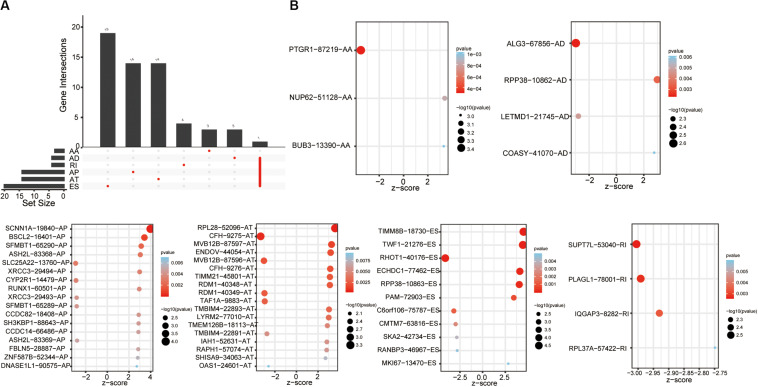
Survival-associated AS events. **(A)** Upset plot of interactions among the seven types of survival-associated AS events in OSCC. **(B)** Survival-associated AS events for constructing survival prediction models.

**FIGURE 3:**
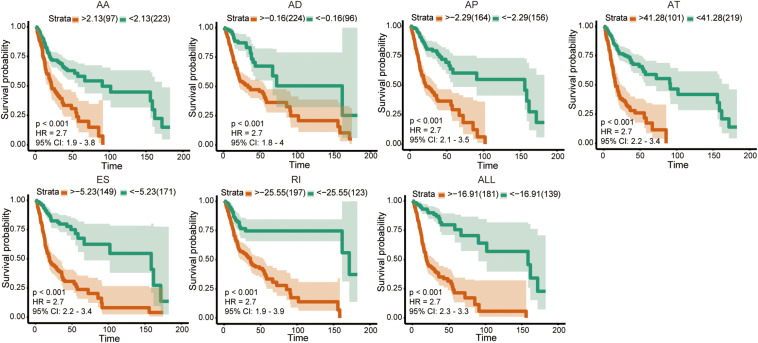
Kaplan–Meier curve of prognostic models in OSCC cohort.

**FIGURE 4:**
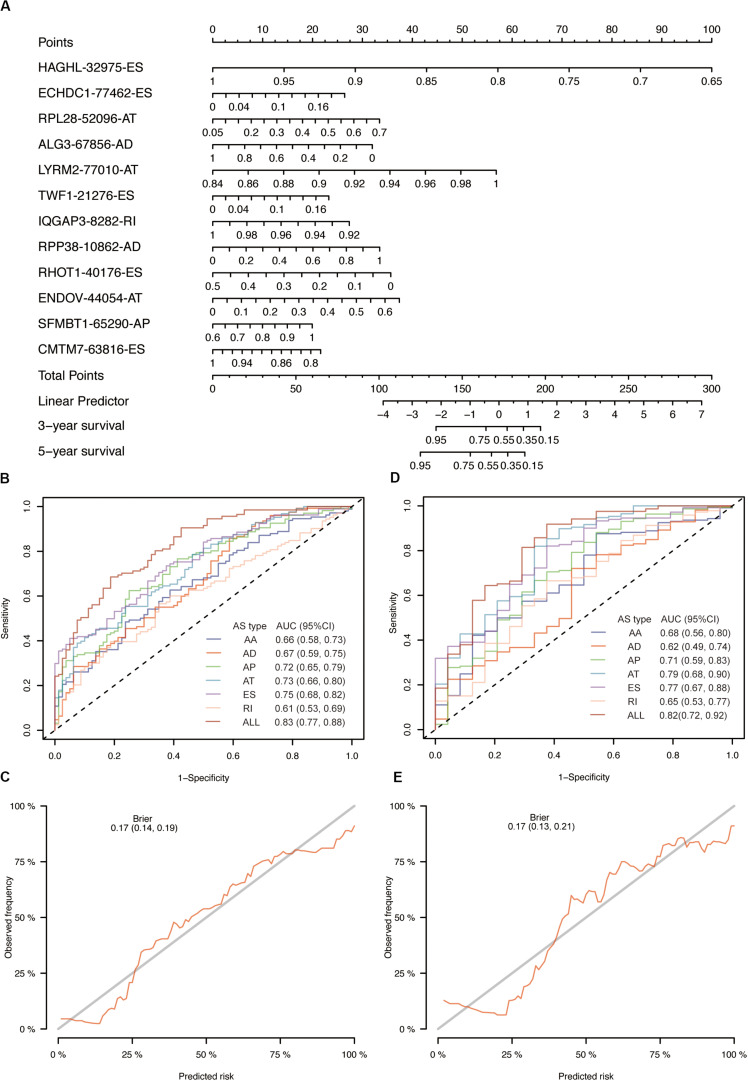
Evaluation of prognostic models in OSCC training cohort. **(A)** The nomogram for predicting probabilities of patients 3-year and 5-year overall survival. **(B)** The ROC curves of seven prognostic models for 3-year overall survival probability. **(C)** The calibration plot of final AS prognostic model for predicting patient 3-year overall survival. **(D)** The ROC curves of seven prognostic models for 5-year overall survival probability. **(E)** The calibration plot of final AS prognostic model for predicting patient 5-year overall survival.

The univariate Cox analysis identified 388 survival-related AS events within 301 genes in our OSCC cohort. After the bootstrapping technique, we finally identified 69 robust prognostic-related AS events within 58 genes. Because no ME events related to survival were found, we did not build predictive models based on MEs. To visualize the interactive sets between six types of AS events, we generated the UpSet plot (Figure 2A). Exon skipping events were selected based on LASSO Cox analysis before multivariate Cox analysis. Figure 2B presented the AS events used in the individual prediction model based on six types of AS. The formulas of theses 6 models were shown in Supplementary Table S2. A new model with more accurate predictive value was established based on the robust prognostic AS events utilizing the method of random forest and multivariate Cox analysis. The total risk score was imputed as follows: -16.42 × HAGHL-32975-ES + 7.597 × ECHDC1-77462-ES + 2.954 × RPL28-52096-AT - 1.837 × ALG3-67856-AD + 20.41 × LYRM2-77010-AT + 7.422 × TWF1-21276-ES - 19.66 × IQGAP3-8282-RI + 1.923 × RPP38-10862-AD - 4.1 × RHOT1-40176-ES + 3.307 × ENDOV-44054-AT + 2.864 × SFMBT1-65290-AP-5.647 × CMTM7-63816-ES. Survival analyses indicated that these prognostic models robustly stratified OSCC patients with different prognosis (Figure 3). Specifically, we observed significantly a shorter OS in high-risk group in all models. Nomogram of 3- and 5-year OS in the OSCC cohort was shown in Figure 4A. ROC analyses were applied to assess the distinguishing ability of prediction models. Within the six separated AS models, the AUC values ranged from 0.61 to 0.75 for 3-year OS and 0.62 to 0.79 for 5-year OS. It is noteworthy that the final model integrating the all types of AS events showed a higher AUC value for 3-year (0.83 [0.77, 0.88]) and 5-year (0.82 [0.72, 0.92]) survival (Figures 4B,D). To assess the agreement between predictive risk and observation risk, we performed the calibration curve. As shown in Figures 4C,E, the final model showed gratifying agreement in the probabilities of 3- and 5-year OS. In addition, the Brier score was 0.17 (0.14, 0.19) for 3-year and 0.17 (0.13, 0.21) for 5-year, indicating a good overall performance in our final model. Furthermore, we also assessed the predictive value of the final model in disease-specific survival (DSS) and relapse-free survival (RFS), which also presented good performances. The AUC was 0.80 [0.73, 0.87] and 0.82 [0.73, 0.92] for 3- and 5-year DDS respectively while that for 3- and 5-year RFS was 0.73 [0.64, 0.82] and 0.76 [0.64, 0.86]. Consistently, the final model showed satisfactory agreement in the probabilities of 3- and 5-year DSS. Besides, when predicting the risk of relapse, the predictive risk was lower than the observed risk (Supplementary Figure S1).

### Internal Validation and Performance

To validate our model, we employed internal validation using bootstrap resampling method (*n* = 1000). Time-dependent AUCs were 0.83 [0.77, 0.89] and 0.82 [0.72, 0.92] for 3- and 5-year survival, respectively. We also observed satisfactory overall performance indicated by Brier score for 3-year (0.17 [0.14, 0.19]) and 5-year (0.17 [0.13, 0.21]) survival. The results of 5-fold cross-validation were similar to bootstrap resampling method (0.83 [0.81, 0.84] and 0.82 [0.76, 0.87] for 3-year and 5-year AUC; 0.17 [0.16, 0.17] and 0.17 [0.15, 0.19] for 3-year and 5-year Brier score) (Table 1).

**TABLE 1:** Model performance in development model and internal validation.

**Measure**	**Final model in development model**	**Final model in internal validation (bootstrap resampling)**	**Final model in internal validation (5 fold cross validation)**
**3-Year overall survival**			
AUC	0.83 [0.77, 0.88]	0.83 [0.77, 0.89]	0.83 [0.81, 0.84]
Brier score	0.17 [0.14, 0.19]	0.17 [0.14, 0.19]	0.17 [0.16, 0.17]
**5-Year overall survival**			
AUC	0.82 [0.72, 0.92]	0.82 [0.72, 0.92]	0.82 [0.76, 0.87]
Brier score	0.17 [0.13, 0.21]	0.17 [0.13, 0.21]	0.17 [0.15, 0.19]

### Risk Score of the AS Signature Was Associated With OSCC Mortality

The results of the Cox analysis were shown in Table 2. When risk score served as a continuous variable, it was closely correlated with the incidence of mortality (2.70 [2.25, 3.25], *P* < 0.0001 in model I; 2.68 [2.22, 3.24], *P* < 0.0001 in model II). Compared with low risk patients, those with high risk had a significantly higher mortality (6.16 [3.85, 9.84], *P* < 0.0001 in model I; 5.90 [3.66, 9.52], *P* < 0.0001 in model II). To verify the robust relevance between risk score and mortality of OSCC, we performed a stratified multivariate Cox analysis and the results were shown in Table 3. The risk score was positively associated with mortality of OSCC in each subgroup (age, sex, grade, and stage) while no interactions were found.

**TABLE 2:** Relationship between risk score and overall survival of OSCC.

**Outcome**	**Crude Model**		**Model I**		**Model II**	
	**HR (95%)**	***P*-value**	**HR (95%)**	***P*-value**	**HR (95%)**	***P*-value**
Risk score	2.72 (2.26, 3.27)	<0.0001	2.70 (2.25, 3.25)	<0.0001	2.68 (2.22, 3.24)	<0.0001
**Risk score**						
Low risk	Reference		Reference		Reference	
High risk	6.15 (3.85, 9.82)	<0.0001	6.16 (3.85, 9.84)	<0.0001	5.90 (3.66, 9.52)	<0.0001

*Model I adjusted for age and sex. Model II adjusted for age, sex, grade and stage.*

**TABLE 3:** Effect size of risk score and overall survival of OSCC in each subgroup.

**Characteristic**	**No. of participants**	**HR (95%CI)**	***P*-value**	***P* for interaction**
**Age (year)**				0.49
<60	141	2.98 (2.05, 4.34)	<0.0001	
≥60	179	2.58 (2.04, 3.25)	<0.0001	
**Sex**				0.37
Male	220	2.84 (2.18, 3.71)	<0.0001	
Female	100	2.46 (1.84, 3.71)	<0.0001	
**Grade**				0.71
G1 + G2	245	2.59 (2.05, 3.27)	<0.0001	
G3 + G4	67	3.29 (2.08, 5.20)	<0.0001	
**Stage**				0.74
Stage I + Stage II	72	2.19 (1.45, 3.31)	<0.0001	
Stage III + Stage IV	219	2.85 (2.25, 3.61)	<0.0001	

*Adjusted for age, sex, grade and stage except the subgroup variable.*

### More Sensitivity to Chemotherapies for Group With Low-Risk

**FIGURE 5:**
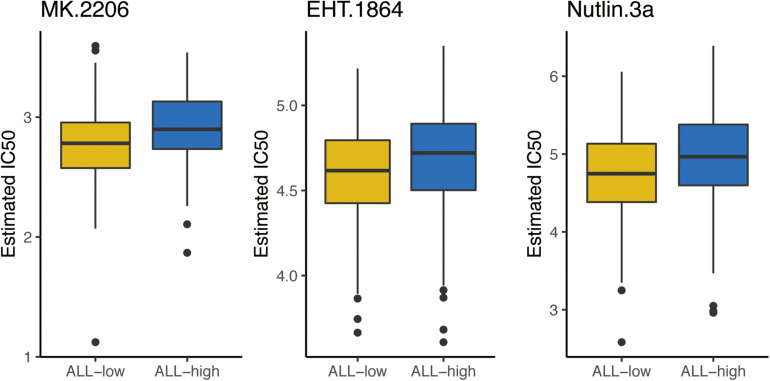
The box plots of the estimated IC50 for 3 chemo drugs between high risk and low risk patients. ALL-L, low risk patients; ALL-H, high risk patients.

According to the results of the final AS model, the included patients could be divided into two subtypes with high risk or low risk. In light of the frequently use of chemotherapy in the treatment of OSCC, we further explored the response of patients with different risk to 138 kinds of chemotherapeutic drugs. In detail, the R package “pRRophetic” and the GDSC cell line data set were combined to facilitate the prediction of IC50 of the selected drugs for every included patient from the TCGA data set. A total of 3 drugs (MK.2206, EHT.1864 and Nutlin.3a) demonstrated obviously lower IC50 in the low-risk group (Figure 5).

### Network Construction of Survival-Associated AS Events

**FIGURE 6:**
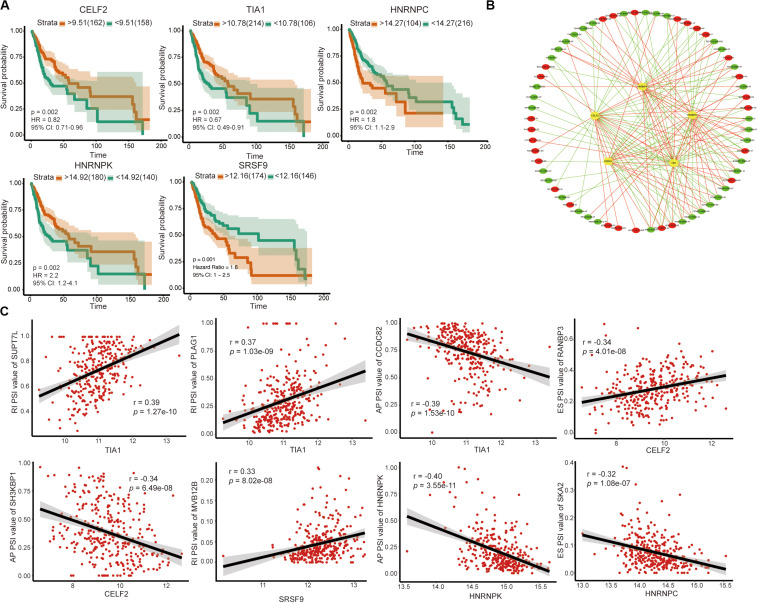
Survival-associated SFs and splicing correlation network in OSCC. **(A)** Kaplan–Meier curve of SFs. **(B)** Splicing correlation network in OSCC; Five survival-associated SFs (yellow dots) were positively (red lines) or negatively (green lines) correlated with AS genes, which predicted favorable (red dots) or adverse (green dots) prognosis. **(C)** Representative dot plots of correlations between the expression of 5 SFs and PSI values of survival-associated AS events.

Among the 71 SFs, we identified 5 survival-related SFs (Figure 6A). Low expression of CELF2 and TIA1 tended to a poor prognosis, whereas high expression of HNRNPC, HNRNPK and SRSF9 indicated a poor prognosis. As expected, we found that the expression of SFs was significantly relevant to the PSI values of 27 AS events with favorable prognosis (red dots) and 35 AS events with poor prognosis (green dots) (Figure 6B). Figure 6C illustrated the representative relationships between SFs and survival-related AS events. For instance, the level of TIA1 increased as RI of SUPT7L or RI of PLAG1 raised but decreased as AP of CCDC82 ascended.

## Discussion

Under the era of precision medicine, notable advances have been made in constructing prognostic models that possess high accurate and sensitivity, and these have facilitated the design of a more individual treating program for patients. Not surprisingly, many OSCC prognostic models were developed. However, the performance of these models could still be improved. In this study, we developed an AS-based model with a moderate predictive ability (AUC = 0.83 and 0.82 for 3- and 5-year OS in the development model; AUC = 0.83 and 0.82 for 3- and 5-year in the bootstrap validation model and 5-fold cross-validation model). Moreover, this model showed good consistency between predicted risk and observed risk, and it is a robust risk factor of OSCC mortality.

A previous study also develops an AS-based prognostic model with good performance (AUC = 0.891 and 0.70 for training and validation cohort, respectively) (20). However, the TCGA OSCC cohort is randomly and equally split into the training and the validation cohort in this former analysis, which is not recommended in the TROPORT statement (21). For one thing, this approach could not develop models based on all available data so that it is statistically inefficient. For another thing, different random splits have different results, which lead to the problem of “replication instability” (15). In addition, this previously established model contains 17 AS based on 165 patients, which cannot meet the vital rule of “EPV 1 to 10” explained above for developing reliable prediction models. Besides, their study also lacks the analysis of another indispensable indicator for model evaluation, namely the calibration curve. Consequently, in view of the above problems, here we adopted the bootstrapping technique since it is the preferred method for internal validation. Moreover, we applied random forest to reduce the number of variables for the purpose of meeting the “EPV 1 to 10 rule of thumb”. Under such methodology improvements, our final model demonstrated little overfitting and satisfactory consistency in the probabilities of 3- and 5-year OS. Yet further external validation of our model is still necessary.

The essential roles of AS in driving tumorigenesis and progression depend on the productions of functional specificity as well as protein diversity and unbalances in AS involved tumor growth, invasion, metabolism and immunity (5, 22, 23). In addition, AS events are associated with the sensitivity of tumors to chemo drugs. Therefore, we explored the sensitivity of patients to 138 chemicals in high-risk and low-risk groups. As expected, OSCC with low risk was more sensitive to three screened drugs.

AS could be regulated by key SFs, making key SFs become the potential therapeutic targets with a good chance (24). To further understand the mechanism of OSCC and identify potential biomarkers as well as therapeutic targets, we also performed a systematic analysis of AS and SFs in OSCC. We identified five survival-related SFs (CELF2, TIA1, HNRNPC, HNRNPK, and SRSF9) and visualized the relationship between the five SFs and 62 survival-related AS events.

CELF2 is an RNA-binding protein of the CELF family that acts as a tumor suppressor and is positively correlated with the prognosis of various tumors, such as non-small cell lung carcinoma (25), gastric cancer (26), and pancreatic cancer (27). The main biological functions of CELF2 are promoting apoptosis as well as inhibiting proliferation and migration We found in this analysis that patients with high expression of CELF2 had a better prognosis, in line with those previous studies. TIA1, T-cell intracellular antigen-1, is another SF with a favorable prognosis we identified. However, its functions may depend on tumor location. For example, TIA1 inhibits progression of gastric cancer by suppressing tumor cell proliferation and accelerating apoptosis (28), whereas it serves as an oncogene in esophageal squamous cell carcinoma through promoting cell proliferation (29).

We also identified three SFs (HNRNPC, HNRNPK, and SRSF9) implying poor prognosis. HNRNPC, heterogeneous nuclear ribonucleoprotein C1/C2, is a commonly expressed RNA-binding protein with cancer-promoting function. Silencing of HNRNPC can inhibit migratory and invasive activities of glioblastoma (30), as well as cell proliferation and tumorigenesis of breast cancer (31). HNRNPK, heterogeneous nuclear ribonucleoprotein K, is frequently upregulated in several kinds of tumors and associated with poor prognosis (32–34). In addition, HNRNPK is bound up with the recurrence of HNSCC (35). SRSF9, serine/arginine-rich SF, serves as an oncogene involved in diverse biological processes, including tumor cell proliferation, apoptosis, migration, and invasion (36–38). On account of the previously studies, the five SFs play important roles in the genesis and development of tumor. Moreover, we found that the five SFs were associated with prognosis-related AS events. Thus, it is reasonable to speculate that dysregulated SFs will promote the occurrence of survival-related AS events, thereby affecting the prognosis of patients.

Within the limits of our study, we developed an AS-based signature with satisfactory performance, identified five key survival-related SFs, and build a prognosis-related SF-AS network. However, further validation of this AS signature in cohort study based on different populations is still needed. Furthermore, the function of key SFs and related regulatory network also requires more exploration and verification *in vitro* and *in vivo*.

## Conclusion

Taken together, a novel AS-based signature with satisfactory performance in risk stratification for OSCC patients was established and low-risk patients tended to be more sensitive to the three chemo drugs. Besides, five key SFs might involve in tumor initiation and progression through regulating the corresponding AS events, offering new alternatives for potential prognosis biomarkers and therapeutic targets.

## Data Availability Statement

Publicly available datasets were analyzed in this study. The datasets used during the current study are available from the TCGA SpliceSeq tool (http://bioinformatics.mdanderson.org/TCGASpliceSeq/) and The Cancer Genome Atlas (TCGA) dataset (https://portal.gdc.cancer.gov/).

## Author Contributions

JX and BC designed the study. RC performed all the bioinformatics analysis described here. RC and JZ wrote and edited the manuscript. YW, LJ, and XR collected and examined the data. JX and BC supervised the project. All authors read and approved the final manuscript.

## Conflict of Interest

The authors declare that the research was conducted in the absence of any commercial or financial relationships that could be construed as a potential conflict of interest.

## Footnotes

1 http://bioinformatics.mdanderson.org/TCGASpliceSeq/

2 https://www.cancerrxgene.org/

3 www.introni.it/spliceaid.html

4 https://tcga-data.nci.nih.gov/tcga/

## Supplementary Material

FIGURE S1 Evaluation of the final AS prognostic model in predicting DDS and RFS. **(A)** The ROC curves of the final AS prognostic model for 3-year and 5-year DDS probability. **(B)** The ROC curves of the final AS prognostic model for 3-year and 5-year RFS probability. **(C,D)** The calibration plot of final AS prognostic model for predicting patient 3-year and 5-year DDS. **(E,F)** The calibration plot of final AS prognostic model for predicting patient 3-year and 5-year RFS.

TABLE S1 Differently AS events in OSCC.

TABLE S2 The formula of 6 type of AS events.
