# Supplementary figures and images for: Comprehensive Analysis of Prognostic Alternative Splicing Signatures in Oral Squamous Cell Carcinoma
Source: Front Oncol. 2020 Aug 28;10:1740. doi: 10.3389/fonc.2020.01740 (PMC7485395; doi:10.3389/fonc.2020.01740)

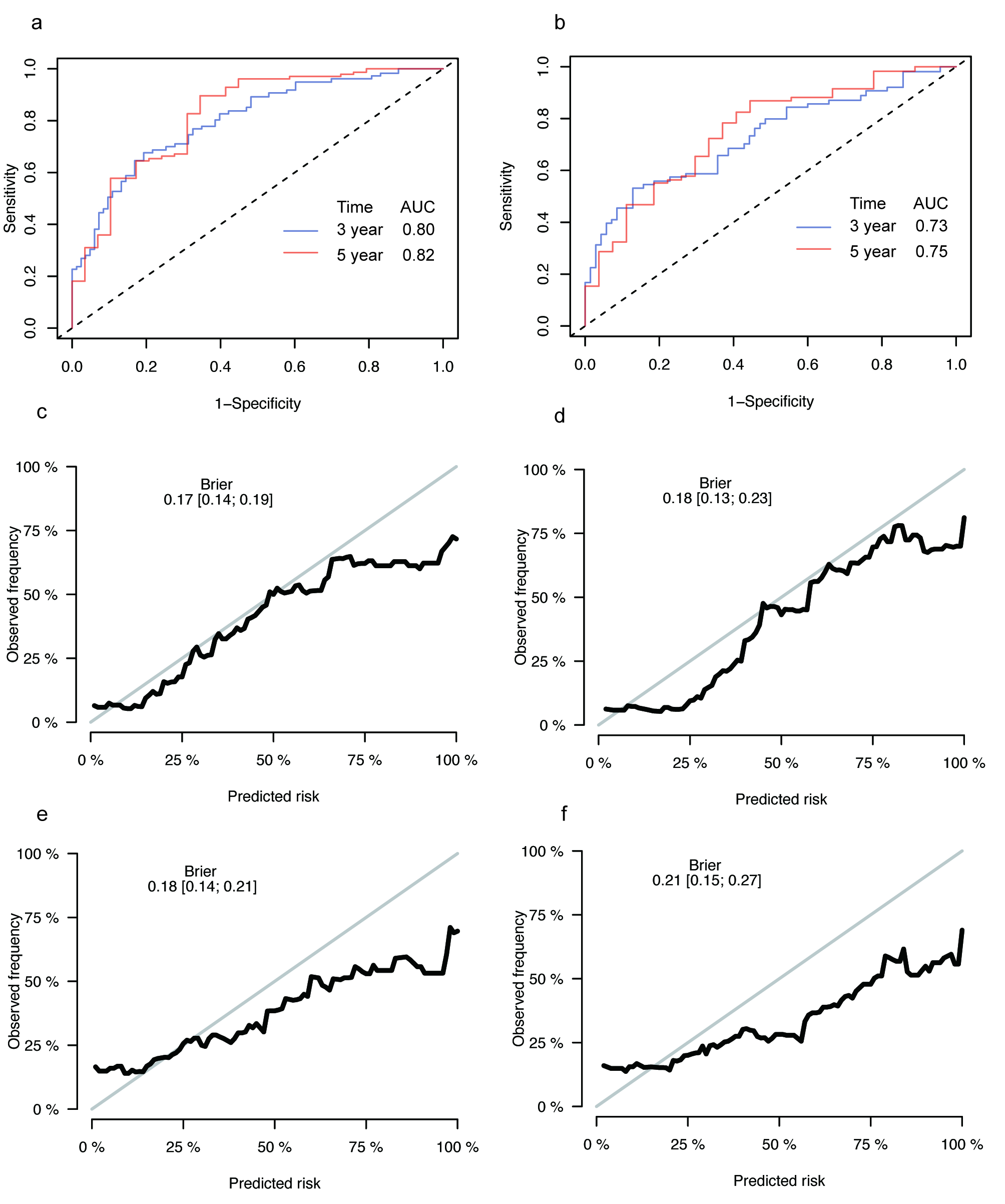

Supplement: FIGURE S1 — Evaluation of the final AS prognostic model in predicting DDS and RFS. (A) The ROC curves of the final AS prognostic model for 3-year and 5-year DDS probability. (B) The ROC curves of the final AS prognostic model for 3-year and 5-year RFS probability. (C,D) The calibration plot of final AS prognostic model for predicting patient 3-year and 5-year DDS. (E,F) The calibration plot of final AS prognostic model for predicting patient 3-year and 5-year RFS. [file Image_1.TIF]
