# Supplementary material for: Comprehensive Analysis of Prognostic Alternative Splicing Signatures in Oral Squamous Cell Carcinoma
Source: Front Oncol. 2020 Aug 28;10:1740. doi: 10.3389/fonc.2020.01740 (PMC7485395; doi:10.3389/fonc.2020.01740)
Supplement: TABLE S1 — Differently AS events in OSCC. [file Table_1.DOCX]

**The formula of 6 type of AS events:**

AA: -5.434011*PTGR1-87219-AA + 1.734038*NUP62-51128-AA + 3.446239* BUB3-13390-AA

AD: -1.79067*ALG3-67856-AD + 1.38085*RPP38-10862-AD - 3.05436*LETMD1-21745-AD+ 0.96901*COASY-41070-AD

AP: 4.365*BSCL2-16401-AP - 5.602*SFMBT1-65290-AP + 1.844*ASH2L-83368-AP - 1.953*SLC25A22-13760-AP - 4.969*CYP2R1-14479-AP + 1.554* RUNX1-60501-AP - 9.554*SFMBT1-65289-AP + 1.739*FBLN5-28887-AP + 2.409*ZNF587B-52344-AP

AT: 2.309*RPL28-52096-AT + 2.696*ENDOV-44054-AT - 5.009*MVB12B-87596-AT + 1.699*CFH-9276-AT + 15.93*TIMM21-45801-AT + 0.7481*RDM1-40348-AT + 17.60*LYRM2-77010-AT - 2.241*TMBIM4-22891-AT + 12.75*SHISA9-34063-AT - 22.15*OAS1-24601-AT

RI: -0.8778*SUPT7L-53040-RI - 1.178*PLAGL1-78001-RI - 23.80*IQGAP3-8282-RI - 0.9908*RPL37A-57422-RI

ES: 3.301*TIMM8B-18730-ES + 11.03*TWF1-21276-ES - 3.431*RHOT1-40176-ES + 2.078*RPP38-10863-ES + 2.611*PAM-72903-ES - 5.566*C6orf106-75787-ES -4.039*CMTM7-63816-ES - 5.539*SKA2-42734-ES - 1.830RANBP3-46967-ES + 1.828*MKI67-13470-ES
